# An enhanced dual IDW method for high-quality geospatial interpolation

**DOI:** 10.1038/s41598-021-89172-w

**Published:** 2021-05-10

**Authors:** Zhanglin Li

**Affiliations:** 1 Computer School, China University of Geosciences, Wuhan, 430074 China; 2 Hubei Key Laboratory of Intelligent Geo-Information Processing, China University of Geosciences, Wuhan, 430074 China

**Keywords:** Environmental sciences, Hydrology

## Abstract

Many geoscience problems involve predicting attributes of interest at un-sampled locations. Inverse distance weighting (IDW) is a standard solution to such problems. However, IDW is generally not able to produce favorable results in the presence of clustered data, which is commonly used in the geospatial data process. To address this concern, this paper presents a novel interpolation approach (DIDW) that integrates data-to-data correlation with the conventional IDW and reformulates it within the geostatistical framework considering locally varying exponents. Traditional IDW, DIDW, and ordinary kriging are employed to evaluate the interpolation performance of the proposed method. This evaluation is based on a case study using the public Walker Lake dataset, and the associated interpolations are performed in various contexts, such as different sample data sizes and variogram parameters. The results demonstrate that DIDW with locally varying exponents stably produces more accurate and reliable estimates than the conventional IDW and DIDW. Besides, it yields more robust estimates than ordinary kriging in the face of varying variogram parameters. Thus, the proposed method can be applied as a preferred spatial interpolation method for most applications regarding its stability and accuracy.

## Introduction

Spatial interpolation (SI) or spatial prediction is a crucial topic in geosciences and related fields such as geology^1,2^, geography^3–5^, hydrology^6,7^, environment^8–11^, and agriculture^12^. To address various concerns in these disciplines, a series of SI methods are developed, which differ in interpolation objectives and basics^13,14^.

Nevertheless, no matter what kinds of contexts are being faced, enhancing the estimation accuracy and reliability is a common goal that most SI methods pursue, and so does the typical SI method—inverse distance weighting (IDW)^1,5,15–21^. In general, the interpolation accuracy of the conventional IDW or its variants could be improved by choosing a set of appropriate parameters such as the search model of local samples or observed data^3,22–24^, the type of distance metric^19,25,26^, and the exponent imposed on the distance^7,22,23,27,28^. One exception is that such parameters are not available for traditional IDW when an uneven sampling rule (which is commonly used in geosciences) is the dominant factor that leads to its low-accuracy estimates. The reason caused this exception is that classical IDW omits the data-to-data relationship.

To overcome this drawback, a modified version of the traditional IDW, dual IDW (DIDW), is proposed in our previous study^29^. By incorporating the D-D correlation into classical IDW, DIDW achieves appropriate estimates in the presence of clustered data. Specifically, DIDW takes into account two kinds of distances: (1) the data-to-data (D-D) distance among local sample data participating in the estimation; and (2) the data-to-unmeasured (D-U) distance from local samples to the location being estimated. Accordingly, two exponents are employed to adjust the relative influence of these two distances on DIDW estimation.

**Figure 1:**
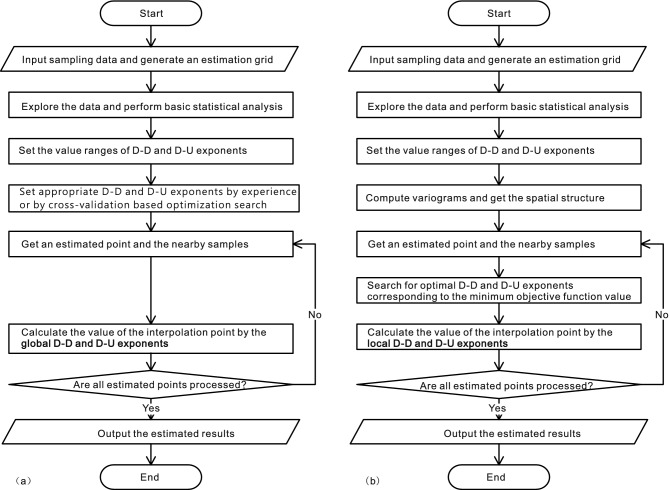
Flowcharts of the traditional DIDW with globally constant exponents (**a**) and the enhanced version with locally varying exponents (**b**).

Despite these merits above, the traditional DIDW^29^ suffers from the invariance of its exponents across the study area and a lack of a practicable criterion for evaluating and finding appropriate DIDW exponents, leading to its limited ability to generate high-quality estimates. Thus, this study proposes an enhanced framework of DIDW with locally varying exponents (LVEs) that significantly improves the interpolation process's flexibility, with enough rationality in accounting for local spatial data configuration and its relationship to the estimated point. To obtain appropriate LVEs, a generalized objective function is developed, which is implemented based on the estimation error variance commonly used in geostatistics^1,30^. The main flowcharts of the traditional and improved DIDW methods are shown in Fig. 1. Compared to globally constant exponents used in the traditional DIDW, LVEs are appropriately incorporated and optimized in the proposed method.

Three methods comprising the traditional IDW with LVEs (IDW-L), DIDW with two global exponents (DIDW-GG), and ordinary kriging (OK) are applied to evaluate the interpolation performance of the proposed method. This evaluation is based on a case study using the public Walker Lake dataset^1^, and the associated interpolations are performed in various contexts, such as different sample data sizes and variogram parameters. Our results demonstrate that the DIDW with LVEs stably produces more accurate estimates than IDW-L and DIDW-GG; it also yields more robust estimates than OK in the face of varying variogram parameters.

The major contributions of this research can be summarized as follows: (1) traditional DIDW is reformulated to incorporate locally varying exponents; (2) the appropriate exponents for each estimated location are adaptively determined using a generalized objective function; and (3) the performance evaluation of the proposed method is also elaborated, confirming its feasibility and stability. Thus, DIDW with LVEs can be applied as a preferred SI method for most applications regarding its stability and accuracy.

## Methods

In this section, traditional DIDW-GG is first introduced. Its improved versions, DIDW with two locally varying exponents (DIDW-LL) and the simplified DIDW-LL (SDIDW-LL), are proposed and elaborated in detail. A brief introduction to OK is illustrated in Supplementary Method online.

### DIDW-GG

Let ${\mathbf{x}}_{i} \left( {i = 1,2, \ldots ,{\text{N}}} \right)$ be a coordinate point in ${\text{q}}$ $\left( {{\text{q}} \ge 1} \right)$ dimensional space and $z\left( {{\mathbf{x}}_{i} } \right)$ be the sampled (observed) value of a variable $z$ at this location. For an unsampled point ${\mathbf{x}}_{0}$ to be estimated, the widely used linear regression estimator $\hat{z}\left( {{\mathbf{x}}_{0} } \right)$ is defined as^1,30^:

1 $$\hat{z}\left( {{\mathbf{x}}_{0} } \right) = \sum\limits_{i = 1}^{{n({\mathbf{x}}_{0})}} {\left[ {\lambda_{i} \left( {{\mathbf{x}}_{0} } \right)z\left( {{\mathbf{x}}_{{\text{i}}} } \right)} \right]}$$

with

2 $$\sum\limits_{i = 1}^{{n({\mathbf{x}}_{0})}} {\left[ {\lambda_{i} \left( {{\mathbf{x}}_{0} } \right)} \right]} = 1$$

where $\lambda_{i} \left( {{\mathbf{x}}_{0} } \right)$ is the estimation weight assigned to the *i*-th measured value $z\left( {{\mathbf{x}}_{i} } \right)$, and $n\left( {{\mathbf{x}}_{0} } \right)$ represents the number of data closest to the estimated location ${\mathbf{x}}_{0}$.

For DIDW-GG, its estimation weight is calculated by^29^:

3 $$\lambda_{i} \left( {{\mathbf{x}}_{0} } \right) = \frac{{d_{0i}^{{ - p_{1} }} \sum\limits_{j = 1}^{{n({\mathbf{x}}_{0} )}} {d_{ij}^{{p_{2} }} } }}{{\sum\limits_{i = 1}^{{n({\mathbf{x}}_{0} )}} {\left[ {d_{0i}^{{ - p_{1} }} \sum\limits_{j = 1}^{{n({\mathbf{x}}_{0} )}} {d_{ij}^{{p_{2} }} } } \right]} }}$$

where $d_{0i}^{{}}$ is the D-U distance from the *i*-th data to the estimated location ${\mathbf{x}}_{0}$; $d_{ij}^{{}}$ represents the D-D distance between the *i*-th and *j*-th sample locations; and $p_{1}$ ($p_{1} \ge 0$) and $p_{2}$ ($p_{2} \ge 0$ ) are the corresponding D-U and D-D exponents to adjust the contributions of $d_{0i}^{{}}$ and $d_{ij}^{{}}$ to the estimation, respectively.

Note that in the case of $p_{2} = 0$, DIDW-GG degrades into the traditional IDW-G, of which the estimation weight is:

4 $$\lambda_{i} ({\mathbf{x}}_{0} ) = \frac{{d_{0i}^{{ - {\text{p}}_{1} }} }}{{\sum\limits_{i = 1}^{{n({\mathbf{x}}_{0} )}} {\left[ {d_{0i}^{{ - {\text{p}}_{1} }} } \right]} }}$$

It is also notable that both D-U and D-D exponents in Eq. (3) are global constants across the study region. This feature may limit DIDW-GG to produce high-quality estimates, especially when the spatial phenomenon under study is involved and the sampling data is irregularly distributed.

### DIDW-LL

Aiming to integrate locally varying exponents in the estimation, each DIDW-GG exponent in Eq. (3) is interpreted as a function of the location being estimated. As a result of this interpretation, the DIDW-LL weight is calculated as follows:

5 $$\lambda_{i} ({\mathbf{x}}_{0} ) = \frac{{d_{0i}^{{ - {\text{p}}_{1} ({\mathbf{x}}_{0} )}} \sum\limits_{j = 1}^{{n({\mathbf{x}}_{0} )}} {d_{ij}^{{{\text{p}}_{2} ({\mathbf{x}}_{0} )}} } }}{{\sum\limits_{i = 1}^{{n({\mathbf{x}}_{0} )}} {\left[ {d_{0i}^{{ - {\text{p}}_{1} ({\mathbf{x}}_{0} )}} \sum\limits_{j = 1}^{{n({\mathbf{x}}_{0} )}} {d_{ij}^{{{\text{p}}_{2} ({\mathbf{x}}_{0} )}} } } \right]} }}$$

where ${\text{p}}_{1} ({\mathbf{x}}_{0} )$ and ${\text{p}}_{2} ({\mathbf{x}}_{0} )$ are the local exponents that can be applied to adjust the contributions of $d_{0i}^{{}}$ and $d_{ij}^{{}}$, respectively.

To a large extent, the two locally varying exponents in Eq. (5) entail the flexibility and suitability of the improved DIDW. For an estimated point surrounded by a set of highly clustered local samples, a large D-D exponent (i.e., ${\text{p}}_{2} ({\mathbf{x}}_{0} )$) should be adopted to produce significant declustering weights. Conversely, if this point is close to a group of regularly distributed samples, a relatively small D-D exponent is preferred to avoid such a strong declustering effect.

Similarly, in the case of ${\text{p}}_{2} ({\mathbf{x}}_{0} ) = 0$, DIDW-LL in Eq. (5) degrades into the traditional IDW-L^23^, of which the estimation weight can be expressed as:

6 $$\lambda_{i} ({\mathbf{x}}_{0} ) = \frac{{d_{0i}^{{ - {\text{p}}_{1} ({\mathbf{x}}_{0} )}} }}{{\sum\limits_{i = 1}^{{n({\mathbf{x}}_{0} )}} {\left[ {d_{0i}^{{ - {\text{p}}_{1} ({\mathbf{x}}_{0} )}} } \right]} }}$$

Besides, if ${\text{p}}_{1} ({\mathbf{x}}_{0} )$ and ${\text{p}}_{2} ({\mathbf{x}}_{0} )$ were constant for every estimated location, Eqs. (5) and (3) would be equal; in other words, DIDW-LL degrades into DIDW-GG in this situation.

### SDIDW-LL

As compared with IDW-L, the flexibility of DIDW-LL is at the cost of complexity. Thus, the estimation weights in Eq. (5) are simplified by assuming that ${\text{p}}_{1} ({\mathbf{x}}_{0} )$ equals ${\text{p}}_{2} ({\mathbf{x}}_{0} )$, resulting in the SDIDW-LL estimation weights:

7 $$\lambda_{i} ({\mathbf{x}}_{0} ) = \frac{{d_{0i}^{{ - {\text{p}}_{1} ({\mathbf{x}}_{0} )}} \sum\limits_{j = 1}^{{n({\mathbf{x}}_{0} )}} {d_{ij}^{{{\text{p}}_{1} ({\mathbf{x}}_{0} )}} } }}{{\sum\limits_{i = 1}^{{n({\mathbf{x}}_{0} )}} {\left[ {d_{0i}^{{ - {\text{p}}_{1} ({\mathbf{x}}_{0} )}} \sum\limits_{j = 1}^{{n({\mathbf{x}}_{0} )}} {d_{ij}^{{{\text{p}}_{1} ({\mathbf{x}}_{0} )}} } } \right]} }}$$

where ${\text{p}}_{1} ({\mathbf{x}}_{0} )$ is the local exponent to simultaneously adjust the influences of $d_{0i}^{{}}$ and $d_{ij}^{{}}$ to the estimation.

### Determination of locally varying exponents

Suppose ${\mathbf{p}}$ is a vector consisting of DIDW-LL exponents to be optimized (e.g., ${\mathbf{p}} = \left[ {{\text{p}}_{1} ({\mathbf{x}}_{0} ),{\text{p}}_{2} ({\mathbf{x}}_{0} )} \right]^{{\text{T}}}$), and ${\text{O}}_{{\text{L}}} \left( {\mathbf{p}} \right)$ is the objective function to evaluate the suitability of these parameters. Then, the corresponding optimization of the local exponents is:

8 $${\mathbf{p}}^{*} = \mathop {\arg \min }\limits_{{{\mathbf{p}} \in {\mathbf{D}}}} \left\{ {{\text{O}}_{{\text{L}}} \left( {\mathbf{p}} \right)} \right\}$$

where ${\mathbf{D}}$ is the definition domain of the vector ${\mathbf{p}}$, and ${\mathbf{D}} \subset {\mathbb{R}}^{{2}}$.

The objective function could be implemented in terms of different assessment criteria, such as the typical error measurements (i.e., true error, absolute error, and so on), interpolation selection index^31^, estimation error variance^1,30,32^, and the intensity of neighboring data^28^. Among these measurements, the error variance is frequently employed in geostatistical methods^23,33^ and considered in this research.

According to the statistical theory on random function model^1^, all of the data $z({\mathbf{x}}_{i} )$ could be interpreted as a realization of the random variable (RV) $Z({\mathbf{x}}_{i} )$. Likewise, this interpretation of the unknown value $z({\mathbf{x}}_{0} )$ and measured value $z({\mathbf{x}}_{i} )$ as realizations of the RVs $Z({\mathbf{x}}_{0} )$ and $Z({\mathbf{x}}_{i} )$ allows one to define the estimation error as an RV, $\left[ {\hat{Z}({\mathbf{x}}_{0} ) - Z({\mathbf{x}}_{0} )} \right]$. Under the stationarity assumption, the estimation error variance can be calculated by^23,30^:

9 $$\begin{gathered} {\text{O}}_{{\text{L}}} \left( {\mathbf{p}} \right) = {\text{Var}} \left\{ {\hat{Z}({\mathbf{x}}_{0} ;{\mathbf{p}}) - Z({\mathbf{x}}_{0} )} \right\} \\ = {\text{C}} \left( 0 \right) - 2\sum\limits_{i}^{{n({\mathbf{x}}_{0} )}} {\lambda_{i} ({\mathbf{x}}_{0} ;{\mathbf{p}}){\text{C}} \left( {{\mathbf{x}}_{i} - {\mathbf{x}}_{0} } \right) + } \sum\limits_{i}^{{n({\mathbf{x}}_{0} )}} {\sum\limits_{j}^{{n({\mathbf{x}}_{0} )}} {\lambda_{i} ({\mathbf{x}}_{0} ;{\mathbf{p}})\lambda_{j} ({\mathbf{x}}_{0} ;{\mathbf{p}}){\text{C}} \left( {{\mathbf{x}}_{i} - {\mathbf{x}}_{j} } \right)} } \\ \end{gathered}$$

where ${\text{C}} \left( \cdot \right)$ stands for the covariance function model used for the study area.

Note that $\lambda_{i} ({\mathbf{x}}_{0} )$ and $\hat{Z}({\mathbf{x}}_{0} )$ are expressed as $\lambda_{i} ({\mathbf{x}}_{0} ;{\mathbf{p}})$ and $\hat{Z}({\mathbf{x}}_{0} ;{\mathbf{p}})$ in Eq. (9), respectively. This expression is to explicitly indicate that the DIDW-LL estimate and weight are related to the parameter vector ${\mathbf{p}}$. Based on Eqs. (8) and (9), the optimized exponents can be rewritten as:

10 $${\mathbf{p}}^{*} = \mathop {\arg \min }\limits_{{{\mathbf{p}} \in {\mathbf{D}}}} \left\{ {{\text{C}} \left( 0 \right) - 2\sum\limits_{i}^{{n({\mathbf{x}}_{0} )}} {\lambda_{i} ({\mathbf{x}}_{0} ;{\mathbf{p}}){\text{C}} \left( {{\mathbf{x}}_{i} - {\mathbf{x}}_{0} } \right) + } \sum\limits_{i}^{{n({\mathbf{x}}_{0} )}} {\sum\limits_{j}^{{n({\mathbf{x}}_{0} )}} {\lambda_{i} ({\mathbf{x}}_{0} ;{\mathbf{p}})\lambda_{j} ({\mathbf{x}}_{0} ;{\mathbf{p}}){\text{C}} \left( {{\mathbf{x}}_{i} - {\mathbf{x}}_{j} } \right)} } } \right\}$$

The parameter vector ${\mathbf{p}}$ in this optimization process is flexible to be specified. For example, it can contain only the D-D or D-U exponent, or both. In this research, three typical application scenarios are chosen as follows:

1. DIDW with locally varying D-U and D-D exponents (i. e., DIDW-LL). In this way, both D-D and D-U exponents are locally optimized in Eq. (10);
2. SDIDW with locally varying D-U and D-D exponents (i. e., SDIDW-LL). The two exponents are equal for SDIDW-LL, and thus only one element needs to be placed in the vector being optimized;
3. DIDW with a local D-U exponent and a global D-D exponent (i. e., DIDW-LG). In this situation, the local D-U exponent is optimized in Eq. (10), while the global D-D exponent can be determined by minimizing cross-validated estimation error.

### Algorithm implementations

The pseudocodes of DIDW-LL and DIDW-LG are described in Algorithm 1 and 2, respectively. It is worth noting that it is necessary to search for an appropriate global D-D exponent based on cross-validation before DIDW-LG is performed.
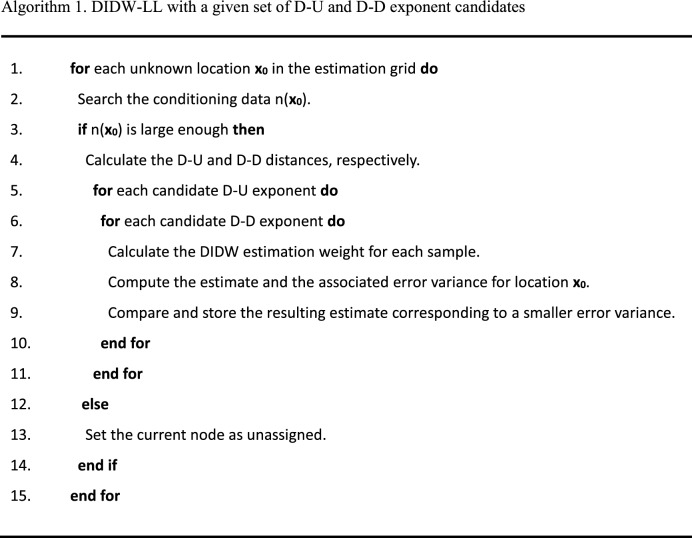



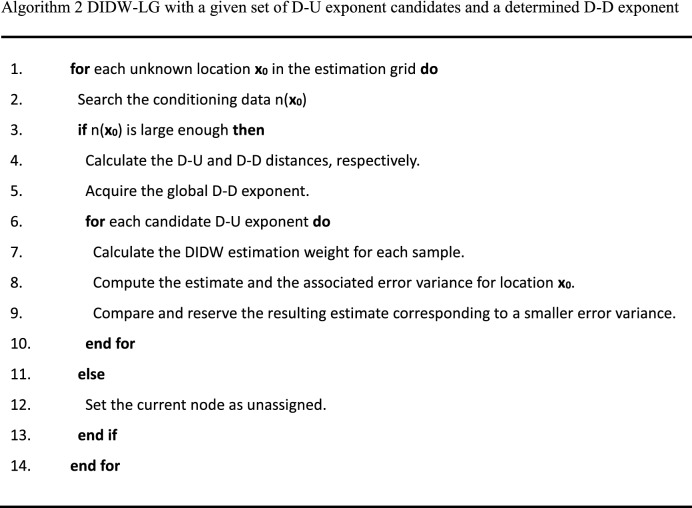


## Results

### Experiment design

For the sake of consistency and comparability between this research and our previous work on DIDW-GG^29^, similar experiment data and calculation parameters to that work are adopted in this study.

### Experiment data

The standard Walker Lake dataset^1,29^ is employed in this research, which is derived from a digital elevation model (DEM) from the western United States, the Walker Lake area in Nevada. Following the interpolation applications in^1^, 470 irregularly spaced samples and 780 regularly distributed locations from this dataset are used as sampled and estimated data, respectively. The origin of the 780 regular points is 5E, 5 N (i.e., X = 5 m, Y = 5 m), and the spacing between points is 10 m in both the north–south and the east–west directions.

**Figure 2:**
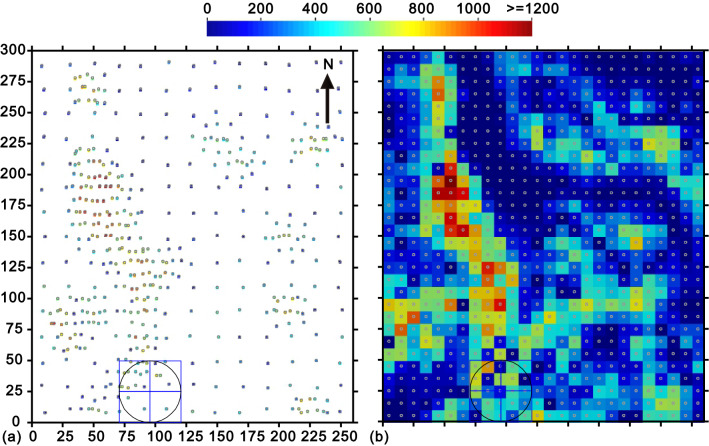
(**a**) 470 sample data and (**b**) 780 grid nodes (10 m by 10 m) to be estimated and their actual values^29^. The sample configuration within the marked search circle is illustrated in Fig. 5.

The locations and the associated attribute values are shown in Fig. 2, along with the complete data in Supplementary Data online. An extensive description of the dataset can be found by^1^.

### Experiment methods

The conventional IDW-L and DIDW-GG are used as benchmarks to assess the interpolation performance of the proposed method. Also, since OK possesses the same optimization objective as DIDW-LL and IDW-L, it is applied as a reference to accomplish the performance evaluation.

Accordingly, there are six methods to be evaluated: DIDW-LL, SDIDW-LL, DIDW-LG, DIDW-GG, IDW-L, and OK. These methods are applied to estimate the 780 grid nodes using the 470 irregular sample points (Fig. 2); their estimates are then compared with the actual values to generate reliable estimation errors. To distinguish it from cross-validated interpolation, this process of interpolating the 780 grid nodes is referred to as "actual interpolation" in the following test.

### Experimental parameters

A series of D-U and D-D exponents ranged from 0.0 to 20.0 with step 0.1, are considered to exhibit the interpolation behavior of the developed methods. Given these exponent candidates, DIDW-LL, SDIDW-LL, DIDW-LG, and IDW-L search for appropriate ones using Eq. (10); DIDW-GG finds its suitable exponents by a cross-validation-based optimization^29^.

**Figure 3:**
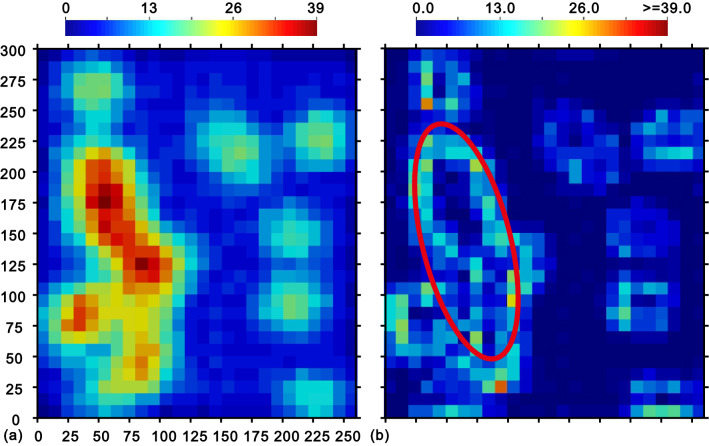
(**a**) Local sample numbers, and (**b**) the variance of the numbers of neighborhood samples in the four quadrants.

All local samples within 25 m are chosen to participate in the estimations. Besides, to observe the clustering feature of neighborhood samples, the available data are divided into quadrants, and the variance of the number of samples in the four quadrants could be used as an index of clustering^1^. Note that the reliability of these indices depends on the total number of conditioning data within each neighborhood (in Fig. 3a); an index resulting from a large number of local samples is more reliable than that with a small sample size. Therefore, the sub-region highlighted by the red ellipse in Fig. 3b is of higher reliability than other locations under study.

To obtain the covariance coefficients in Eq. (10), N14°W is chosen as the direction of maximum continuity, and its variogram adopted is^1^:

11 $$\gamma_{\max } \left( h \right) = \left\{ {\begin{array}{*{20}c} {0 \, } & {{\text{if}}\;h = 0} \\ {22,000 + 40,000{\text{Sph}}_{30} \left( h \right) + 45,000{\text{Sph}}_{150} \left( h \right)} & {{\text{if}}\;h > 0} \\ \end{array} } \right.$$

In the direction of minimum continuity (N76°E), the model is:

12 $$\gamma_{\min } \left( h \right) = \left\{ {\begin{array}{*{20}c} {0 \, } & {{\text{if}}\;h = 0} \\ {22,000 + 40,000{\text{Sph}}_{25} \left( h \right) + 45,000{\text{Sph}}_{50} \left( h \right)} & {{\text{if}}\;h > 0} \\ \end{array} } \right.$$

**Figure 4:**
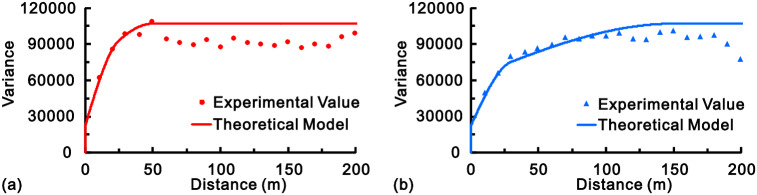
The experimental variograms and their fitted models in the directions of minimum (**a**) and maximum (**b**) continuity.

The accompanying experimental and theoretical variograms in these two directions are shown in Fig. 4.

### An illustration of DIDW-LL weights

**Figure 5:**
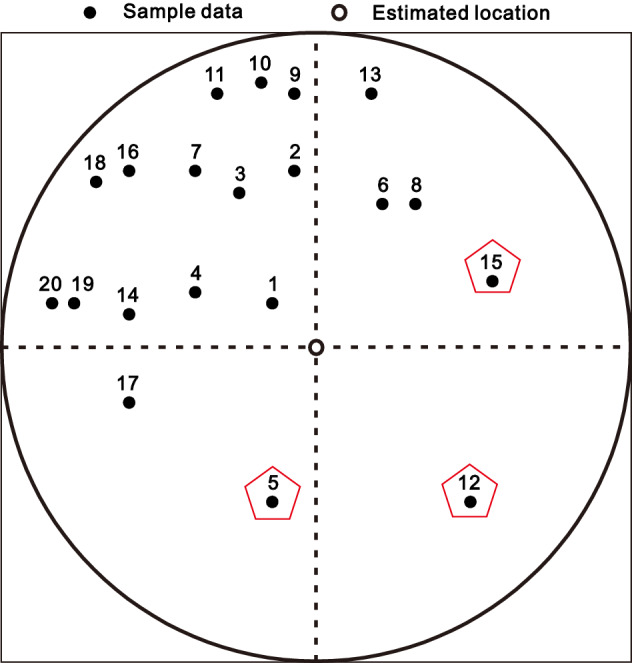
The typical estimation instance marked in Fig. 2. Note that the pentagons highlight the three samples with the least redundancies.

**Figure 6:**
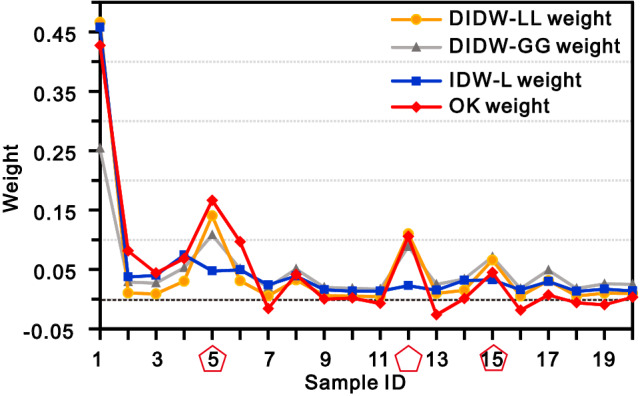
Resulting weights associated with the data pattern shown in Fig. 5 using the four different estimators. For the DIDW-GG, its D-D and D-U exponents are both set as 2.0.

A representative estimation instance corresponding to the sample configuration marked by the search circle in Fig. 2 is depicted in Fig. 5. The associated DIDW-LL, DIDW-GG, IDW-L, and OK estimation weights are illustrated in Fig. 6. Some observations can be made about this figure.

First, IDW-L yields unreasonable sample weights with respect to data redundancy. For example, this approach does not recognize the relative importance of the samples indicated by the pentagons in Fig. 5. In contrast, DIDW-LL, DIDW-GG, and OK reasonably account for the underlying data redundancy in this sample configuration.

Besides, the resulting weights from DIDW-LL and OK are quite similar due to the same estimation objective, implying that DIDW-LL would approximate OK in terms of estimates and the associated error variances. This phenomenon for DIDW-LL is reasonable and expectable since kriging's underlying declustering mechanism is widely accepted^1,34^. On the other hand, DIDW-GG does not bear such a significant resemblance to OK, especially for the first data point (i.e., the sample with an ID of "1") in Fig. 6. It should be pointed out that, by tuning its D-D and D-U exponents, DIDW-GG could account for a specific data configuration satisfactorily. However, it may be difficult for DIDW-GG to search for very suitable D-D and D-U exponents simultaneously for multiple estimated points because its exponents are constant across the study area. Further analyses on the correlation between OK and DIDW-LL, DIDW-LG, IDW-L are illustrated in the following sections.

Moreover, note that the negative OK weights can be observed. Although these weights are valid and acceptable in theory, they would also lead to unrealistic estimates in some practical applications^35^. Noticeably, this issue will not arise in the developed methods as the basic idea of weight assignment of IDW is inherited by DIDW.

Consequently, DIDW-LL has favorable characteristics in the following aspects: (1) compared with IDW-L, it can recognize the clustered sample data more accurately; (2) relative to OK, it entails non-negative estimation weights; and (3) as compared with DIDW-GG, it has more opportunities to appropriately account for the sample configuration regarding every single estimated point.

### DIDW-LL and SDIDW-LL estimations

**Figure 7:**
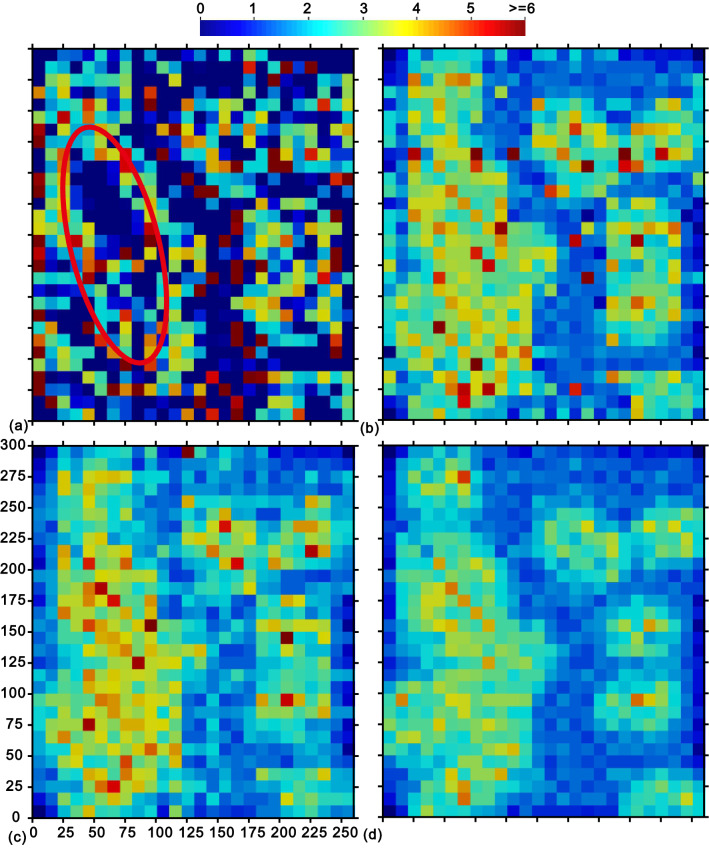
(**a**) D-D and (**b**) D-U exponents of DIDW-LL; D-U exponents of (**c**) SDIDW-LL and (**d**) IDW-L.

As stated above, all of the test estimators are applied to interpolate the 780 grid nodes (in Fig. 2). Figure 7a exhibits the D-D exponents resulting from the DIDW-LL estimation. As expected, they are overall in line with the clustering degree of local data represented in Fig. 3b, especially for the highlighted elliptical sub-area. Generally, the more strong clustering is observed, the larger D-D exponents will be.

Figure 7b–d represents the corresponding D-U exponents from the DIDW-LL, SDIDW-LL, and IDW-L methods, respectively. They have similar spatial distribution patterns to the local data numbers shown in Fig. 3a. The overall feature is that the estimated locations with a large number of conditioning data tend to be attached with a high D-U exponent; conversely, a relatively low D-U exponent is applied when the number of local samples is small.

**Figure 8:**
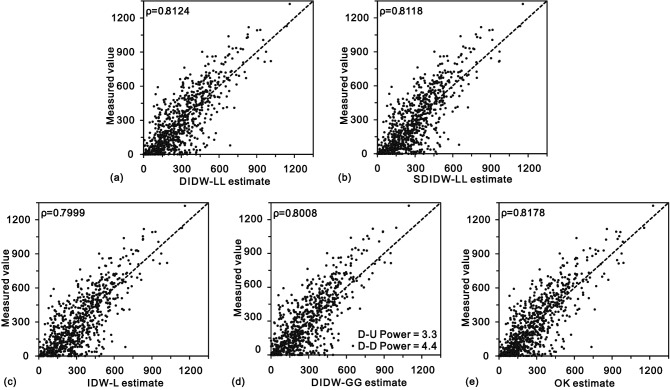
Scatterplots of the measured values vs. estimates from (**a**) DIDW-LL; (**b**) SDIDW-LL; (**c**) IDW-L, (**d**) DIDW-GG, and (**e**) OK.

**Figure 9:**
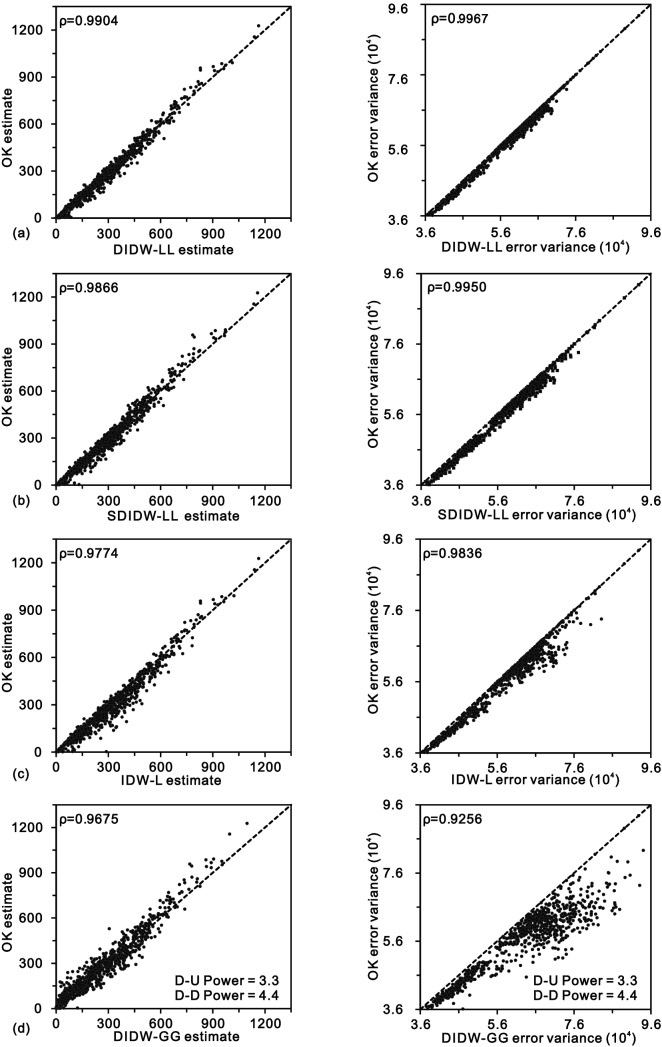
Comparisons between estimates and error variances from OK and those from (**a**) DIDW-LL; (**b**) SDIDW-LL; (**c**) IDW-L and (**d**) DIDW-GG.

Figure 8 depicts the comparisons of the actual values and estimates from DIDW-LL, SDIDW-LL, and the reference estimators (IDW-L, DIDW-GG, and OK). DIDW-LL, SDIDW-LL, and OK possess very similar interpolation accuracy, superior to either IDW-L or DIDW-GG. The scatterplots represented are similar to each other, especially for the variogram-based estimators (i.e., DIDW-LL, SDIDW-LL, IDW-L, and OK). This feature is further exhibited in Fig. 9, which indicates that the estimates and the associated error variances from DIDW-LL and SDIDW-LL bear a more significant correlation to the OK results than those from IDW-L and DIDW-GG. This phenomenon is expectable because IDW-L ignores the D-D correlation, and DIDW-GG does not aim to minimize the estimation error variance.

Consequently, DIDW-LL and SDIDW-LL produce very similar estimates and error variances to OK; both estimators are superior to the traditional IDW-L and DIDW-GG concerning the flexibility, interpolation accuracy, and the ability to produce a lower estimation error variance.

### DIDW-LG estimation

To evaluate the interpolation performance of DIDW-LG, cross-validation is first applied to determine an appropriate global D-D exponent, which is then employed to accomplish the interpolation for the 780 estimated locations.

#### Cross-validations

**Figure 10:**
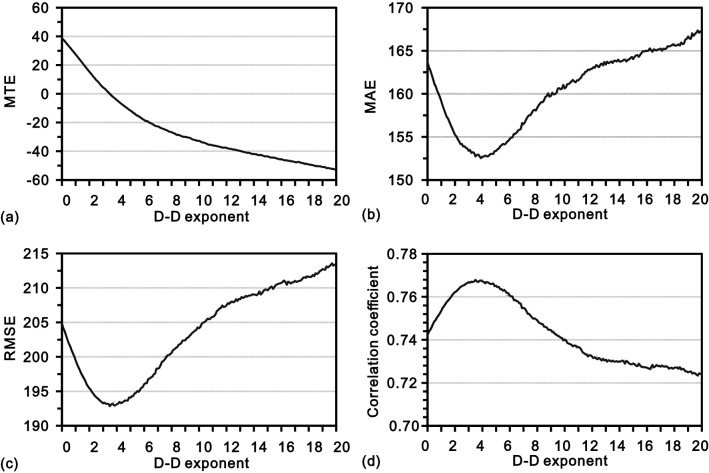
Cross-validation based interpretation accuracies from DIDW-LG with varying D-D exponents. (**a**) MTE; (**b**) MAE; (**c**) RMSE; and (**d**) correlation coefficient.

In the process of cross-validation using DIDW-LG, four classical error measurements, including mean true error (MTE), mean absolute error (MAE), root mean square error (RMSE), and the correlation coefficient between actual and estimated values, are used to explore the interpolation accuracy as well as to determine an appropriate global D-D exponent. The corresponding results are shown in Fig. 10, and some observations can be made as follows.

First, in Fig. 10a, as the D-D exponent increases, the MTE presents a monotonic decreasing tendency, indicating a continuous decrease of the associated estimates in total. This decline of the estimates, resulting from the declustering, is in line with the sampling strategy (the samples are preferentially collected in the high-value areas as shown in Fig. 2a) and thus demonstrates the validity of DIDW-LG.

Additionally, it is also notable that the origin of each subplot in Fig. 10 corresponds to the case when IDW-L is used. Obviously, there are numerous D-D exponents, which would entail that DIDW-LG is more accurate than IDW-L.

Moreover, both MAE and RMSE indicate that a D-D exponent of 4.0 is appropriate, thus employed in the actual interpolation below.

#### Actual interpolations

**Figure 11:**
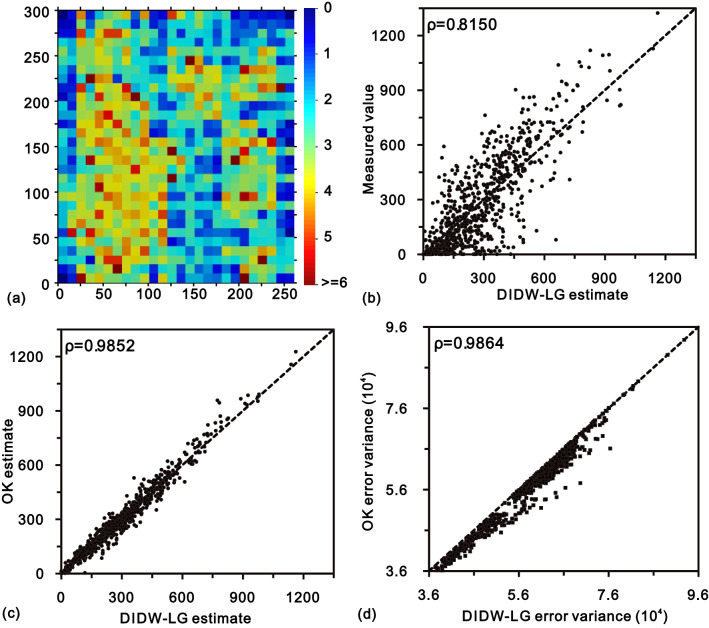
(**a**) Optimized D-U exponents from DIDW-LG; comparisons of DIDW-LG estimate with (**b**) actual value and (**c**) OK estimate; and (**d**) scatterplot of error variances from DIDW-LG and OK.

Based on the optimal D-D exponent stated above, the actual interpolation using DIDW-LG is conducted, and the corresponding results are depicted in Fig. 11. Overall, the essential characteristics of DIDW-LG results, including the D-U exponents, interpolation accuracy, and the similarity compared with OK, are consistent with DIDW-LL and SDIDW-LL (shown in Fig. 7). This consistency demonstrates that DIDW-LG also produces more favorable estimates than IDW-L and DIDW-GG.

**Figure 12:**
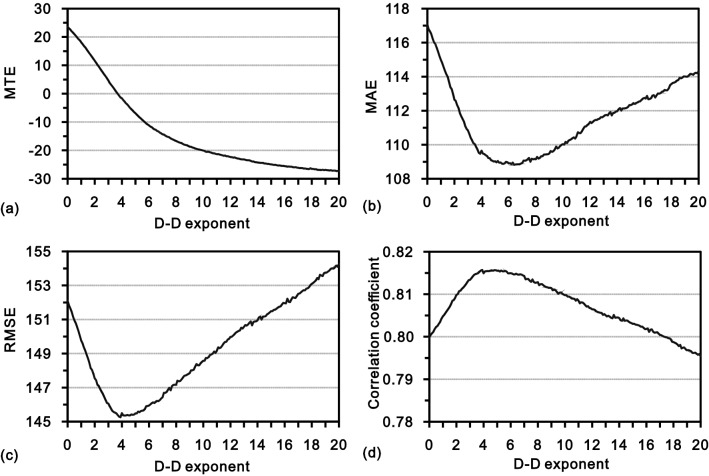
Interpretation accuracies associated with the estimates from DIDW-LG with varying D-D exponents. (**a**) MTE; (**b**) MAE; (**c**) RMSE; and (**d**) correlation coefficient.

Moreover, it is still worth providing qualitative insights into the actual interpolation performance of DIDW-LG with different D-D exponents. In Fig. 12, it can be observed that the behavior of MTE from DIDW-LG is normal as expected, which is rather similar to what is revealed in Fig. 10a. Likewise, as exhibited by RMSE or MAE, there are numerous D-D exponents that would yield more accurate DIDW-LG estimates than the conventional IDW-L.

### Sensitivity analysis

In this section, a series of different sample datasets and spatial correlation parameters are generated to test the reliability and stability of the developed methods.

#### Test with different datasets

Ten sample sub-datasets, drawn as 10%, 20%, …, 100% of the data from the 470 sample points and orderly named as S10, S20, …, S100, are applied to estimate the 780 grid nodes by the tested estimators. The detailed sample locations of these datasets can be found as Supplementary Fig. S1 online.

**Figure 13:**
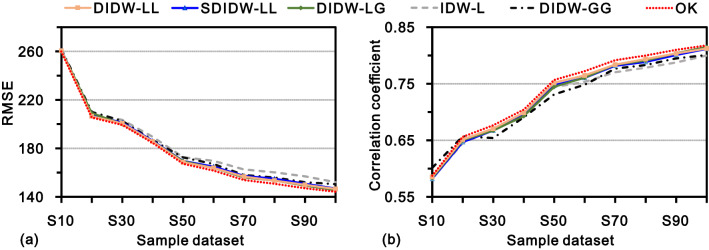
Actual interpolation accuracies of the tested methods with varying sample datasets, measured by (**a**) RMSE and (**b**) correlation coefficient.

As exhibited in Fig. 13 and its accompanying result in Table 1, in general, IDW-L produces the most inaccurate results among the test methods. The main reason should be that IDW-L completely ignores the correlation among sample data. On the contrary, OK yields the most accurate estimates. Following OK, DIDW-LL and DIDW-LG yield very similar estimation results, which are slightly more accurate than SDIDW-LL. Despite this, SDIDW-LL is still superior to either IDW-GG or IDW-L with respect to interpolation accuracy.

**Table 1:** RMSE and correlation coefficient (CC) corresponding to various estimators using different sample datasets.

Dataset	DIDW-LL		SDIDW-LL		DIDW-LG		IDW-L		DIDW-GG		OK	
	RMSE	CC	RMSE	CC	RMSE	CC	RMSE	CC	RMSE	CC	RMSE	CC
S10	260.49	0.5854	260.96	0.5817	260.63	0.5834	261.30	0.5826	261.80	0.6012	259.86	0.5873
S20	206.40	0.6512	207.47	0.6467	209.01	0.6500	208.25	0.6448	209.98	0.6569	205.38	0.6560
S30	200.51	0.6706	201.09	0.6672	200.03	0.6672	203.47	0.6682	202.72	0.6539	199.26	0.6765
S40	185.66	0.6987	186.16	0.6950	185.62	0.6927	189.46	0.6921	186.75	0.6904	184.36	0.7040
S50	168.47	0.7517	169.54	0.7471	169.37	0.7444	172.65	0.7442	172.50	0.7312	167.22	0.7566
S60	163.78	0.7646	164.89	0.7604	163.67	0.7606	169.64	0.7530	167.03	0.7481	161.55	0.7722
S70	156.60	0.7838	157.24	0.7816	155.90	0.7835	162.58	0.7704	158.00	0.7767	153.85	0.7915
S80	153.40	0.7935	154.92	0.7886	153.49	0.7909	160.17	0.7784	155.92	0.7830	150.74	0.7999
S90	149.85	0.8035	150.77	0.8007	149.27	0.8037	156.89	0.7875	152.02	0.7947	147.05	0.8098
S100	146.46	0.8124	146.78	0.8118	145.50	0.8149	152.11	0.7999	150.44	0.8008	144.13	0.8178

These characteristics are generally consistent with those illustrated in the above tests (as shown in Sect. 4.3 and 4.4), implying the stability of the developed methods in the context of various sample datasets.

#### Test with different variogram parameters

It is widely accepted that the practical success of kriging estimators heavily depends on the suitability of the chosen variogram ^36^. Likewise, due to the introduction of the error variance in Eq. (10), either DIDW-LL or DIDW-LG is unavoidably dependent on the reliability of the spatial structure. Nevertheless, the degree of this dependence is not very clear, which deserves to be elaborated.

To achieve this elaboration, the reference variogram model in Eq. (11) is perturbed to generate a set of spatial structures in the following two aspects: (1) ten main anisotropy angles, evenly dividing the search space, are designed based on the main anisotropic direction (340°) of the reference variogram model; (2) likewise, the first range, 30 m, along the direction of maximum continuity in Eq. (11) is applied to create ten new variogram models through equally increasing its value by 0 m, 10 m, 20 m, …, 90 m.

**Figure 14:**
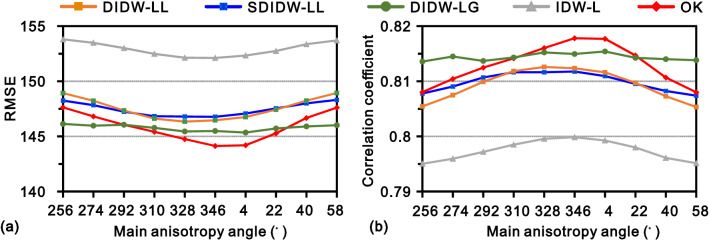
Actual interpolation accuracies of the tested methods with varying anisotropy angles, measured by (**a**) RMSE and (**b**) correlation coefficient.

Figure 14 exhibits the resulting interpolation accuracies of the five variogram-based methods with various anisotropy angles. Judging from the bend degree of the RMSE or correlation coefficient curves, the most sensitive method to the main anisotropy angle is OK, followed by IDW-L, DIDW-LL, and SDIDW-LL, which bear similar sensitivities; DIDW-LG presents significant stability under the condition of various directions of maximum continuity. The tested methods sorted by the overall interpolation accuracy from best to worst are OK, DIDW-LG, DIDW-LL, SDIDW-LL, and IDW-L, respectively. Nevertheless, it is noticeable that the DIDW-LG with several main anisotropy angles, such as 40° and 58°, also generates more accurate estimates than OK.

**Figure 15:**
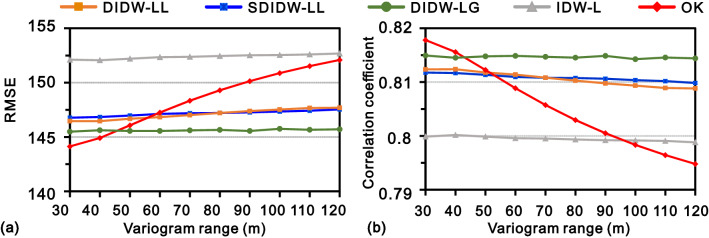
Actual interpolation accuracies of the tested methods with varying variogram ranges, measured by (**a**) RMSE and (**b**) correlation coefficient.

Figure 15 reveals the corresponding estimates in the case of varying variogram ranges. Most methods represent favorable stability except OK, which tends to yield less accurate estimates than IDW-L in terms of the RMSE or correlation coefficient.

Consequently, all three implementations of the proposed DIDW with LVEs (i.e., DIDW-LL, SDIDW-LL, and DIDW-LG) are significantly superior to the traditional IDW-L and DIDW-GG. When the spatial correlation is accurately captured, their results could bear significant similarity to OK outcomes; otherwise, they may outperform OK, especially for DIDW-LG.

## Discussion

To some extent, it is rational to consider that DIDW with LVEs approximates OK since they share the same optimization goal, minimizing estimation error variance. This approximation would be enhanced by using variogram distance instead of the Euclidean metric employed in this study, probably improving the estimation accuracy when spatial anisotropy in the study region is significant. However, this replacement should be cautiously applied since it may increase the dependency of the proposed method on the spatial structure.

Moreover, the designed objective function could be implemented more flexibly. For instance, other estimation parameters in the proposed method, such as the type of search model and search radius, can also be added into the vector ${\mathbf{p}}$ in Eq. (10), and optimized together with the local exponents to further improve the interpolation accuracy. For the sake of practicability, more advanced optimization technologies in machine learning methods, such as the genetic algorithm^37,38^ and simulation annealing^39^, would be helpful to achieve this goal.

Finally, the main characteristics of OK and DIDW with LVEs is summarized in Table 2. In addition to the two methods, the radial basis function interpolation (RBFI)^40,41^ is described in this table, because it is also a frequently used SI method that accounts for the effect of clustering. It is notable that, unlike RBFI and OK, the proposed method does not need to solve a system of equations. This feature would be attractive in a big data or high-dimensional context, where numerical instability of the solution to the system exists.

**Table 2:** Main characteristics of OK, radial basis function interpolation (RBFI), and DIDW with LVEs.

Main characteristics	OK	RBFI	DIDW with LVEs
Is it dependent on statistical theory?	Yes	No	Yes
Does it include a declustering mechanism?	Yes	Yes	Yes
Does it need to solve a system of equations?	Yes	Yes	No

## Conclusions

In this paper, a new dual IDW framework (DIDW with LVEs) that can account for the D-D and D-U correlations flexibly is proposed. It involves two key points: (1) the original DIDW formalism is modified to incorporate the LVEs; (2) a generalized objective function aiming to minimize the estimation error variance is developed to determine appropriate LVEs. Within this framework, DIDW can self-adaptively choose suitable exponents according to local data configuration and correlation. This feature entails that DIDW can capture locally changed physical features, thereby increasing the accuracy and reliability of its estimates.

The real-world application shows that DIDW with LVEs is more flexible and robust than the traditional IDW-L and DIDW-GG. Besides, it is superior to OK in many aspects; for instance, it is immune to negative estimation weights, applicable for high-dimensional SI issues, and less sensitive to variogram parameters.

In future work, the author plans to empower DIDW with enough capabilities in accounting for complex spatial dependency^42–44^ and finding more efficient means to seek appropriate LVEs.

## Supplementary Information

Supplementary Data.

Supplementary Figure S1.

Supplementary Method.

## Abbreviations

D-D: Data to data
D-U: Data to unmeasured/unsampled location
SI: Spatial interpolation
IDW: Inverse distance weighting; a typical SI method only considering D-D distances
DIDW: Dual IDW; an improvement of IDW, simultaneously considering D-D and D-U distances
SDIDW: A simplified DIDW, using the same value for D-U and D-D exponents of DIDW
OK: Ordinary kriging; a typical SI method in geostatistics
LVEs: Locally varying exponents (the exponent of a distance is a crucial parameter of IDW)
IDW-G: IDW with one globally constant D-U exponent
IDW-L: IDW with locally varying D-U exponents
DIDW-GG: DIDW with globally constant D-U and D-D exponents
DIDW-LG: DIDW with one locally varying D-U exponent and one globally constant D-D exponent
DIW-LL: DIDW with locally varying D-U and D-D exponents
SDIDW-LL: SDIDW with locally varying D-U and D-D exponents
