# Supplementary material for: An enhanced dual IDW method for high-quality geospatial interpolation
Source: Sci Rep. 2021 May 10;11:9903. doi: 10.1038/s41598-021-89172-w (PMC8110750; doi:10.1038/s41598-021-89172-w)
Supplement: Supplementary file 2 — Supplementary Figure S1. [file 41598_2021_89172_MOESM2_ESM.pdf]

An enhanced dual IDW method for high-quality geospatial interpolation

Zhanglin Li[a,b]

Corresponding author: Zhanglin Li (lizhl@cug.edu.cn)

[a] Computer School, China University of Geosciences, Wuhan, 430074, China

[b] Hubei Key Laboratory of Intelligent Geo-Information Processing, China University of Geosciences, Wuhan, 430074, China

Fig. S1 reveals the sample locations of the datasets S10-S90, which are used in the ‘Sensitivity Analysis’ section. Note that dataset S100, corresponding to all of the original 470 sample data, is exhibited in Fig. 1a and not shown in this figure.

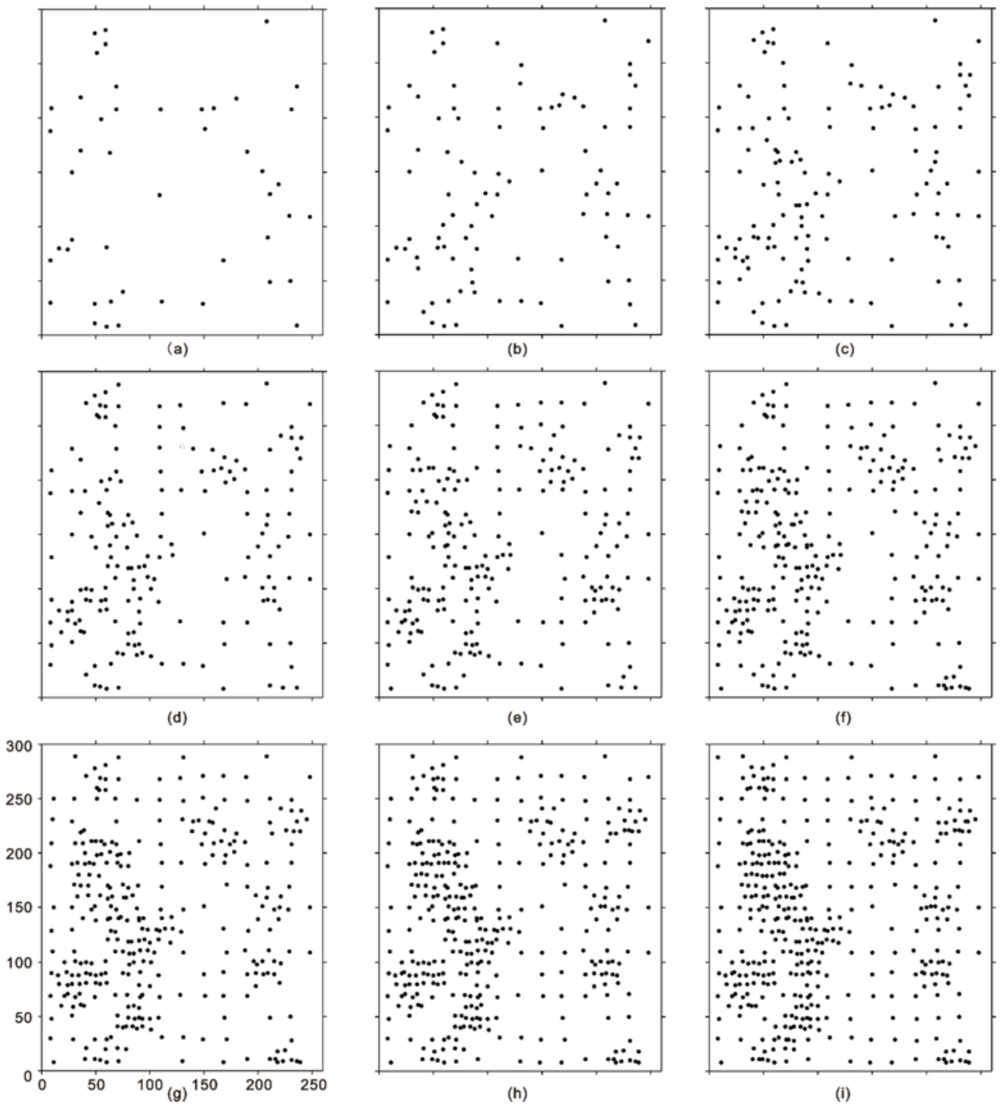

Fig. S1 Locations of the nine sample datasets randomly drawn from the 470 estimated samples: (**a**) S10; (**b**) S20; (**c**) S30; (**d**) S40; (**e**) S50; (**f**) S60; (**g**) S70; (**h**) S80; and (**i**) S90
