# Supplementary material for: An enhanced dual IDW method for high-quality geospatial interpolation
Source: Sci Rep. 2021 May 10;11:9903. doi: 10.1038/s41598-021-89172-w (PMC8110750; doi:10.1038/s41598-021-89172-w)
Supplement: Supplementary file 3 — Supplementary Method. [file 41598_2021_89172_MOESM3_ESM.pdf]

An enhanced dual IDW method for high-quality geospatial interpolation

Zhanglin Li[a,b]

Corresponding author: Zhanglin Li (lizhl@cug.edu.cn)

[a] Computer School, China University of Geosciences, Wuhan, 430074, China

[b] Hubei Key Laboratory of Intelligent Geo-Information Processing, China University of Geosciences, Wuhan, 430074, China

In this text, classical kriging methods [1,2] are briefly introduced in their primal and dual forms.

1. Kriging in the primal form

Consider the estimation of a continuous attribute z at any unsampled location $\mathbf{x}$ using n data $\{z(\mathbf{x}_a), a = 1, \cdots, n\}^{\mathrm{T}}$. In kriging paradigm, $z(\mathbf{x})$ and $z(\mathbf{x}_a)$ are interpreted as the realizations of random variables $\mathrm{Z}(\mathbf{x})$ and $\mathrm{Z}(\mathbf{x}_a)$. Given this interpretation, typical kriging estimate of $\mathrm{Z}(\mathbf{x})$ can be expressed as [1]:

$$\hat{Z}(\mathbf{x}) = m(\mathbf{x}) + \sum_{i=1}^{n(\mathbf{x})} \lambda_i(\mathbf{x})[Z(\mathbf{x}_i) - m(\mathbf{x}_i)] \tag{S1}$$

where $\lambda_i(\mathbf{x})$ is the weight assigned to datum $z(\mathbf{x}_i)$ (which is interpreted as a realization of the random variable $Z(\mathbf{x}_i)$); $n(\mathbf{x})$ is the number of data closest to $\mathbf{x}$, that is, only the $n(\mathbf{x})$ data within a given neighborhood $\mathrm{W}(\mathbf{x})$ centered on $\mathbf{x}$ are used in the estimation; $m(\mathbf{x})$ and $m(\mathbf{x}_i)$ are expected values of the random variables $Z(\mathbf{x})$ and $Z(\mathbf{x}_i)$ [1].

To reasonably acquire the value of $\lambda_i(\mathbf{x})$ in Eq.(S1), all flavors of kriging share the same objective of minimizing the following error variance [3]:

$$\sigma_{\mathrm{E}}^2(\mathbf{x}) = \mathrm{Var}\{\hat{Z}(\mathbf{x}) - Z(\mathbf{x})\} \tag{S2}$$

under the constraint of unbiasedness of the estimator:

$$\mathrm{E}\{\hat{Z}(\mathbf{x}) - Z(\mathbf{x})\} = 0 \quad \text{(S3)}$$

1.1. Kriging with a trend model

Kriging with a trend model (KT) is a typical kind of kriging method, modeling its local mean or trend as a linear combination of functions $f_k(\mathbf{x})$ of the coordinates:

$$m(\mathbf{x}') = \sum_{k=0}^{K} a_k(\mathbf{x}') f_k(\mathbf{x}') \qquad \forall \mathbf{x}' \in \mathrm{W}(\mathbf{x}) \quad \text{(S4)}$$

where the coefficients $a_k(\mathbf{x}')$ are unknown constants within each local neighborhood $\mathrm{W}(\mathbf{x})$.

It follows from Eqs. (S1-4) that [1]:

$$\begin{cases} \sum_{i=1}^{n(\mathbf{x})} \lambda_i(\mathbf{x}) \mathrm{C}_R(\mathbf{x}_j - \mathbf{x}_i) + \sum_{k=0}^{K} \mu_k(\mathbf{x}) f_k(\mathbf{x}_j) = \mathrm{C}_R(\mathbf{x}_j - \mathbf{x}) & j = 1, \cdots, n(\mathbf{x}) \\ \sum_{i=1}^{n(\mathbf{x})} \lambda_i(\mathbf{x}) f_k(\mathbf{x}_i) = f_k(\mathbf{x}) & k = 0, 1, \cdots, K \end{cases} \quad \text{(S5)}$$

where $\mu_k(\mathbf{x})$ are the Lagrange parameters used for deriving this system of equations, and $\mathrm{C}_R(.)$ stands for the covariance function of the residual component of a random variable. For instance, $\mathrm{C}_R(\mathbf{x}_j - \mathbf{x}_i)$ is the covariance of the residual component of $\left(Z(\mathbf{x}_j) - Z(\mathbf{x}_i)\right)$.

Once the estimation weights $\lambda_i(\mathbf{x})$ are obtained by Eq. (S5), the following expression can be used to calculate the corresponding KT estimate:

$$\hat{z}(\mathbf{x}) = \sum_{i=1}^{n(\mathbf{x})} \lambda_i(\mathbf{x}) z(\mathbf{x}_i) \quad \text{(S6)}$$

The associated error variance is:

$$\sigma_{KT}^2(\mathbf{x}) = \mathrm{C}_R(0) - \sum_{i=1}^{n(\mathbf{x})} \lambda_i(\mathbf{x}) \mathrm{C}_R(\mathbf{x}_i - \mathbf{x}) - \sum_{k=0}^{K} \mu_k(\mathbf{x}) f_k(\mathbf{x}) \quad \text{(S7)}$$

1.2. Ordinary kriging

In the case of $f_0(\mathbf{x}') = 1$ and $K = 0$ (i.e., $m(\mathbf{x}')$ *is a constant but unknown mean* a_0), KT is equivalent to ordinary kriging (OK). The corresponding OK system of linear equations is:

$$\begin{cases} \sum_{i=1}^{n(\mathbf{x})} \lambda_i(\mathbf{x})\mathrm{C}(\mathbf{x}_j - \mathbf{x}_i) + \mu_0(\mathbf{x}) = \mathrm{C}(\mathbf{x}_j - \mathbf{x}) & j = 1, \cdots, n(\mathbf{x}) \\ \sum_{i=1}^{n(\mathbf{x})} \lambda_i(\mathbf{x}) = 1 \end{cases} \tag{S8}$$

where $\mathrm{C}(.)$ stands for the covariance function of a random variable. For OK, $\mathrm{C}_R(.) = \mathrm{C}(.)$.

When OK estimation weights $\lambda_i(\mathbf{x})$ are obtained by solving Eq. (S8), its estimate can also be obtained by Eq.(S6), with the following error variance:

$$\sigma_{OK}^2(\mathbf{x}) = \mathrm{C}(0) - \sum_{i=1}^{n(\mathbf{x})} \lambda_i(\mathbf{x})\mathrm{C}(\mathbf{x}_i - \mathbf{x}) - \mu_0(\mathbf{x}) \tag{S9}$$

2. Kriging in dual form

2.1. Dual KT

KT in its dual form (dual KT) uses the following estimation formula:

$$\hat{z}(\mathbf{x}) = \sum_{k=0}^{K} a_k(\mathbf{x}) f_k(\mathbf{x}) + \sum_{i=1}^{n(\mathbf{x})} d_i(\mathbf{x})\mathrm{C}_R(\mathbf{x}_i - \mathbf{x}) \tag{S10}$$

The weights $a_k(\mathbf{x})$ and $d_i(\mathbf{x})$ can be obtained by the following system of equations:

$$\begin{cases} \sum_{i=1}^{n(\mathbf{x})} d_i(\mathbf{x})\mathrm{C}_R(\mathbf{x}_j - \mathbf{x}_i) + \sum_{k=0}^{K} a_k(\mathbf{x}) f_k(\mathbf{x}_j) = z(\mathbf{x}_j) & j = 1, \cdots, n(\mathbf{x}) \\ \sum_{i=1}^{n(\mathbf{x})} d_i(\mathbf{x}) f_k(\mathbf{x}_i) = 0 & k = 0, 1, \cdots, K \end{cases} \tag{S11}$$

2.2. Dual OK

Similarly, dual OK is a particular case of dual KT, (i.e., $f_0(.) = 1$ and $K = 0$), and its estimation

expression is:

$$\hat{z}(\mathbf{x}) = a_0 + \sum_{i=1}^{n(\mathbf{x})} d_i(\mathbf{x})\mathrm{C}(\mathbf{x}_i - \mathbf{x}) \tag{S12}$$

where the weights a_0 and $d_i(\mathbf{x})$ can be obtained by solving:

$$\begin{cases} \sum_{i=1}^{n(\mathbf{x})} d_i(\mathbf{x})\mathrm{C}(\mathbf{x}_j - \mathbf{x}_i) + a_0 = z(\mathbf{x}_j) \quad j = 1, \cdots, n(\mathbf{x}) \\ \sum_{i=1}^{n(\mathbf{x})} d_i(\mathbf{x}) = 0 \end{cases} \tag{S13}$$

3. Main characteristics of kriging

From the above introductions, two notable characteristics related to the research are:

(1) Kriging has considered the data-to-data and data-to-unknown correlations, using the covariance values $\mathrm{C}(\mathbf{x}_j - \mathbf{x}_i)$ and $\mathrm{C}(\mathbf{x}_i - \mathbf{x})$, respectively. DIDW, the main method discussed in the paper, also possesses this feature. Thus, kriging is used as a key benchmark to assess the performance of DIDW.

(2) As clearly shown in its dual form, kriging is almost equivalent to radial basis function (RBF) interpolation[4,5], especially for the dual KT (dual OK can be viewed as a particular case of RBF interpolation). From this respective, the only difference between RBF interpolation and kriging is that, by convention, they usually use different forms of positive definite functions (i.e., covariance functions or RBF kernels) to keep the validity of the solution to the system of equations.

References

1 Goovaerts, P. *Geostatistics for Natural Resources Evaluation*. (Oxford University Press, 1997).

2 Isaaks, E. H. & Srivastava, R. M. *An Introduction to Applied Geostatistics*. (Oxford University Press, 1989).

3 Huijbregts, C. J. & Journel, A. G. *Mining geostatistics*. (Academic Press, 1978).

4 Buhmann, M. D. *Radial Basis Functions: Theory and Implementations*. (Cambridge University Press, 2003).

5 Horowitz, F., Hornby, P., Bone, D. & Craig, M. Fast Multidimensional Interpolations. in *26th Proceedings of the Applications of Computers and Operations Research in the Minerals Industry (APCOM).* (ed R. V. Ramani) 536 (Society for Mining, Metallurgy, and Exploration).
